# Are High-Severity Fires Burning at Much Higher Rates Recently than Historically in Dry-Forest Landscapes of the Western USA?

**DOI:** 10.1371/journal.pone.0136147

**Published:** 2015-09-09

**Authors:** William L. Baker

**Affiliations:** Program in Ecology/Department of Geography, Dept. 3371, 1000 E. University Ave., University of Wyoming, Laramie, Wyoming, United States of America; Ecole Pratique des Hautes Etudes, FRANCE

## Abstract

Dry forests at low elevations in temperate-zone mountains are commonly hypothesized to be at risk of exceptional rates of severe fire from climatic change and land-use effects. Their setting is fire-prone, they have been altered by land-uses, and fire severity may be increasing. However, where fires were excluded, increased fire could also be hypothesized as restorative of historical fire. These competing hypotheses are not well tested, as reference data prior to widespread land-use expansion were insufficient. Moreover, fire-climate projections were lacking for these forests. Here, I used new reference data and records of high-severity fire from 1984–2012 across all dry forests (25.5 million ha) of the western USA to test these hypotheses. I also approximated projected effects of climatic change on high-severity fire in dry forests by applying existing projections. This analysis showed the rate of recent high-severity fire in dry forests is within the range of historical rates, or is too low, overall across dry forests and individually in 42 of 43 analysis regions. Significant upward trends were lacking overall from 1984–2012 for area burned and fraction burned at high severity. Upward trends in area burned at high severity were found in only 4 of 43 analysis regions. Projections for A.D. 2046–2065 showed high-severity fire would generally be still operating at, or have been restored to historical rates, although high projections suggest high-severity fire rotations that are too short could ensue in 6 of 43 regions. Programs to generally reduce fire severity in dry forests are not supported and have significant adverse ecological impacts, including reducing habitat for native species dependent on early-successional burned patches and decreasing landscape heterogeneity that confers resilience to climatic change. Some adverse ecological effects of high-severity fires are concerns. Managers and communities can improve our ability to live with high-severity fire in dry forests.

## Introduction

Wildfires have increased since the 1980s in some parts of the world [1], including parts of the western USA [2–5], but are recent and projected rates of severe fire, that kill most trees, well above historical rates and a threat to forest landscapes? Some dry forests of the temperate zone, which are prone to wildfires, are thought to be experiencing exceptionally high rates or sizes of severe fire relative to historical fires [6]. Earlier springs, warmer temperatures, decreased precipitation, and increased drought consistent with global warming, are contributing to increased fire since the 1980s over substantial area [2, 4]. Increased severe fire in dry forests is also attributed to past land-uses (e.g., logging, livestock grazing), that led to unnatural fuel buildup [7]. However, analysis of fire responses to warming and drying and land-use legacies across multiple regions shows effects can be heterogeneous and even divergent [8–9].

Moreover, upward trends alone do not indicate whether recent rates of severe wildfires are below, near, or above historical rates. The first hypothesis, stated above, is that fire has already reached unprecedented rates in dry forests. An alternative hypothesis is that fires were reduced over the last 1–2 centuries by intentional fire suppression and indirect effects of land uses (e.g., reduction in fine fuels that facilitate fire spread). Thus, increased fire now could be restorative of the rate component of the historical fire process, which is commonly considered an essential part of restoring western dry forests [10]. Managers are allowing more wildfires to burn under controlled conditions to restore fire across dry-forest landscapes [11]. If wildfire is operating within, or being restored to its historical range of variability, then many aspects of the ecology of affected forest landscapes will likely also be functioning as they did historically [12]. However, there are some ecological responses to high-severity fire (e.g., post-fire tree recruitment) that could be hampered by increasing drought and rising temperatures [13]. Whether upward-trending high-severity fire is hypothesized to be restorative of the rate component of the historical fire process or leading to too much high-severity fire depends on the frame of reference and how rates compare. Here I use new evidence from dry forests in the western USA to test these competing hypotheses. I also approximate rates of future severe fire in these dry forests.

### Background on the hypotheses and projected future fire

Support for the hypothesis that rates of recent high-severity fire are exceptionally high relative to historical rates comes in part from recent trends in high-severity fire, traditionally defined as severe fire that kills 75% or more of the basal area in a forest stand [14]. A statistically significant upward trend in area burned at high severity was found over the last few decades in the southern Rockies, on the Colorado Plateau, and in mountainous parts of central and southern Arizona and New Mexico, but not in the northern Rockies or Pacific Northwest [3–4] or in Yosemite National Park in California [15]. Fraction of fire that burned at high severity also increased significantly in the southern Rockies, but not elsewhere [3]. These studies, however, were not specific to dry forests.

In dry forests, a statistically significant upward trend in area burned at high severity was not found in the Eastern Cascades of Oregon and Washington, but was in the Klamath province [14]. Upward trends in area burned at high severity and fraction burned at high severity were initially found for dry forests of the Sierra Nevada, Modoc Plateau, and Southern Cascades [16] and for fraction burned at high severity in northwestern California [5]. Analysis with a more complete dataset for the Sierra Nevada found no trend in area burned or fraction burned at high severity [17]. No significant upward trend was found in fraction burned at high severity in dry provinces in the Pacific Northwest [14]. However, these studies did not generally aim to resolve whether trends have led to historically unprecedented high-severity fire.

Studies over longer periods appear to support the restorative hypothesis, but do not address fire severity, and have incomplete evidence. Area-burned data for the 11 western states qualitatively suggest the 1980s increase could be restorative and a return to the higher rates of burning of the 1910s-1930s [18]. Estimates of pre-1900 area burned, derived using an assumption that composite fire intervals from tree-ring fire histories are equal to fire rotation, suggest so much historical fire that recent trends would definitely be restorative, as almost an order of magnitude more fire would be needed to match historical area burned [19–20]. However, the assumption that composite fire intervals represent fire rotation is not supported [21]. Charcoal data over the last 3,000 years suggest fire closely tracked climate until a peak in the middle-1800s, when a fire deficit began, which may link to landscape fragmentation and fire suppression, but data for the last few decades are unfortunately unresolved in these records [22].

Reconstructions of historical fire with the needed rate estimates for severe fire are rare. The key rate parameter is the fire rotation, the expected time for high-severity fire to burn an area equal to a study area of interest [21]. Most early tree-ring studies assumed severe fire was rare in dry forests, and did not study it [23]. Some recent tree-ring studies reconstructed fire severity and found evidence of historical high-severity fire in dry forests [24], but did not estimate fire rotations. Historical high-severity fire is also documented in dry forests by early maps, photographs, and records [25], but these, too, have not been used to estimate fire rotations. Data from early aerial-photographic research [26] have been used [27]. Recently, we used new methods, based on survey data from the U.S. General Land Office (GLO), to reconstruct historical fire severity and fire rotation in dry forests across large land areas [28]. These data sources [28–30] show high-severity fire occurred historically in all studied dry-forest landscapes, and rates of high-severity fire were modest to low, with fire rotations of centuries (Table 1). These rates for high-severity fire are corroborated by sedimentary charcoal records [31–38], which document episodes of high-severity fire at similar rates (Table 1). The charcoal data are from debris-flow sediments mobilized after heavy precipitation on severe burns.

**Table 1:** Reconstructions of fire rotation (FR) for high-severity fire in historical dry forests of the western USA, with corroborating evidence from sedimentary charcoal studies.

Author(s)	Location	Method^1^	High-severity FR (years) and severe fire-episode intervals^2^
DRY PINE FORESTS			
Baker [29]	E. Cascades, E Oregon	GLO tree data	***705***
DRY MIXED-CONIFER FORESTS			
Baker [30]	W. Sierra Nevada Mts., W California	GLO tree data and line data	***281–354***
Long *et al*. [31]	E. Cascades, E Oregon	Charcoal in sediment deposits	333^2^ ^,^ ^3^
Odion *et al*. [27]	N. Sierra Nevada Mts., W California	Early historical	***488***
Baker [29]	E. Cascades, E Oregon	GLO tree data	***496***
Fitch [32]	Jemez Mts., N New Mexico	Charcoal in sediment deposits	500? (400–667)^2^ ^,^ ^4^
COMBINED DRY PINE AND MIXED-CONIFER FORESTS			
Pierce and Meyer, [33] and Pierce *et al*., [34]	Central Idaho	Charcoal in sediment deposits	(154–286)^2^ ^,^ ^5^
Williams and Baker, [28]	Black Mesa, N Arizona	GLO tree data	***217***
Jenkins *et al*., [35]	Mogollon Plateau, N Arizona	Charcoal in sediment deposits	250 (200–400)^2^
Williams and Baker, [28]	Front Range, E Colorado	GLO tree data	***271***
Odion *et al*., [27]	E Cascades, E Washington	Aerial photos	***379–505***
Baker, [29]	E Cascades, E Oregon	GLO tree data	***435***
Bigio, [36]	San Juan Mts., SW Colorado	Charcoal in sediment deposits	> 471 (> 667)^2^ ^,^ ^6^
Colombaroli and Gavin, [37]	Siskiyou Mts, SW Oregon	Charcoal in sediment deposits	500 (142)^2^
Frechette and Meyer, [38]	Sacramento Mts., SE New Mexico	Charcoal in sediment deposits	500 (667)^2^ ^,^ ^7^
Williams and Baker, [28]	Mogollon Plateau and Black Mesa, N Arizona combined	GLO tree data	***522***
Williams and Baker, [28]	Mogollon Plateau, N Arizona	GLO tree data	***828***
Williams and Baker, [28]	Blue Mts., NE Oregon	GLO tree data	***849***

Studies are arranged by length of the fire rotation. Estimates from GLO data, FIA data, and early aerial photographs are shown in bold italics to emphasize their higher precision, while corroborative, less certain estimates from charcoal records are shown in regular type. The range of estimates in bold is used as the reference in this study.

^1^ Methods for reconstruction included using charcoal data from sediment, using early aerial photographs or historical records, using the GLO tree data and a calibrated model [28]. I did not use the GLO line data’s direct records of entry and exit in burned areas, as these records represent moderate- to high-severity fires, not exclusively high-severity fires [40].

^2^ These are intervals between severe fire episodes evident in alluvial deposits, that could approximate high-severity fire rotations, but are uncertain since area burned is not known and fire severity is more approximately reconstructed than with other methods. I considered data for the last 500 years from each paleo-environmental study, but also included in parentheses the interval between episodes in the last 2000 years, where this is available.

^3^ These authors indicate that it is difficult to determine fire severity from their methods, and only identify the recent fire frequency as 3 per 1000 years, but they indicate that the documented fire episodes were followed by up to 100 years of recovery, which does suggest severe fires, although this is my interpretation.

^4^ Fitch [32] suggested that low-severity fire dominated from 870 cal yr BP, but explains the possibility, but uncertainty, of a severe fire around 400 cal yr BP (p. 40), thus I include this single event, with a question mark, for the 500-year estimate. More certain is evidence of 3–5 severe fires in the last 2000 years (p. 42), but those all preceded 870 cal yr BP.

^5^ These authors were not focused on counting the number of fire-episodes over the last 2000 years, thus I roughly estimated this from Fig 5B in Pierce and Meyer [33] as between 7–13, as there are 7 broad peaks in this figure, but they also report 9 major debris flows between about 950 and 1150 AD, thus the total could reach as many as 13. No severe fires occurred in the last 500 years.

^6^ This author provided data on the number of watersheds, out of six sampled, that burned in high-severity events [36]. I used these data to approximate a high-severity fire rotation using the standard formula: period of observation / fraction of area burned. Thus, for the last 550 years, a total of 7 watersheds burned, thus the fire rotation is 550 / (7/6) = 471 years. And for the last 2000 years, a total of 18 watersheds burned, thus a fire rotation of 667 years. However, Bigio indicates that sample locations may be high in a watershed, thus it is not known that the whole watershed burned. This leaves these estimates as minima, which I have indicated by using “>” before the estimate.

^7^ These authors identify periods of severe-fire activity after c. 1800 cal, yr BP, a peak in 800–500 cal yr BP, and at least one large, severe fire in the last 400 years, thus perhaps 3 episodes in the last 2000 years and one in the last 500 years. However, this is my approximation from their data, as they do not report recurrence intervals for severe fire.

These sources show evidence of historical high-severity fire across a wide spectrum of biophysical settings in dry-forest landscapes, but high-severity fire may have been favored in particular settings, allowing for at least temporary persistence of some dry forests with only low-moderate severity fire [e.g., 28]. Recent high-severity fire in the western USA, not specific to dry forests, often shows preference for higher elevations, steeper topography, and northerly-facing aspects [39]. Similar topographic effects occurred historically in dry forests, but with only modest influence, as higher-severity fires (mixed- and high-severity fire) spanned diverse biophysical settings across 624,000 ha of historical dry forests in the Colorado Front Range [40]. Similarly, extensive reconstructions (303,000 ha) of historical dry forests in the Pacific Northwest suggest open, old-growth forests with low- to moderate-severity fire were favored at lower elevations, on shallower slopes, and on more southerly-facing aspects, but even in these settings “were perhaps ephemeral in nature, lasting one or more centuries at a location, and then switching concordant with regional climate forcing to non-equilibrium states” [26]. Further research is needed about spatial variation in high-severity fire in historical dry forests.

Area burned by wildfire is expected to increase with global warming, adding to unprecedented rates of severe fire, or, alternatively, enhancing restoration of the historical fire process. Global projections showed annual area burned at any severity may reach about 2.8 times current area burned in some regions by A.D. 2100, depending on emissions scenario, but large areas of fire declines may also occur [8]. In the western United States, McKenzie *et al*. [41] projected, using one climate model, that by A.D. 2100, ratios of future to recent area burned could range from < 1.0 in California and Nevada up to about 1.4–2.5 in Arizona, Colorado, Idaho, Montana, and Oregon, about 3 in Washington and Wyoming, and near 5 in New Mexico and Utah. The most recent projections for the western United States used a large ensemble of climate models and a moderate emissions scenario to project fire in A.D. 2046–2065 [42]. They projected ratios of future-to-recent area burned of 1.63–2.24 in the Southwest, 1.71–2.69 in Rocky Mountain Forests, 1.62–2.00 in the Eastern Rocky Mountains/Great Plains, 1.56 times in the Nevada Mountains/Semi-desert, and 1.24 times in a California Coastal Shrub aggregated ecoregion [42]. Models did not agree well for the Pacific Northwest, but ratios were 1.42–2.54. Ranges represent outcomes from two modeling approaches.

## Methods

### Dry forests

Dry forests are the primary mid-elevation forests of the western USA that include dry pine forests and dry mixed-conifer forests. These are roughly sequentially arrayed along an elevation and moisture gradient extending from just above semi-arid woodlands (e.g., piñon-juniper) to just below moist mixed-conifer forests that typically occur near the ecotone with subalpine forests [43]. To define the geographical location and extent of historical dry forests, I used Biophysical Settings (BpS) maps from Landfire [44], a government program to map vegetation, fuels, and related data about fire across the United States [45]. The BpS maps have 30-m resolution pixels from Landsat satellite data, and use biophysical variables to predict vegetation, defined by NatureServe Ecological Systems [46] before widespread expansion of EuroAmerican land uses. BpS maps may avoid the problem of a phantom trend in fire from using maps of vegetation after the beginning of a trend analysis period [17]. This is a concern I will revisit in the discussion. However, the BpS maps have accuracy, as do other Landsat-derived maps, of only about 64–67% for Ecological Systems in forests [47]. A test in Utah found generally lower, but some higher accuracies [48].

**Fig 1:**
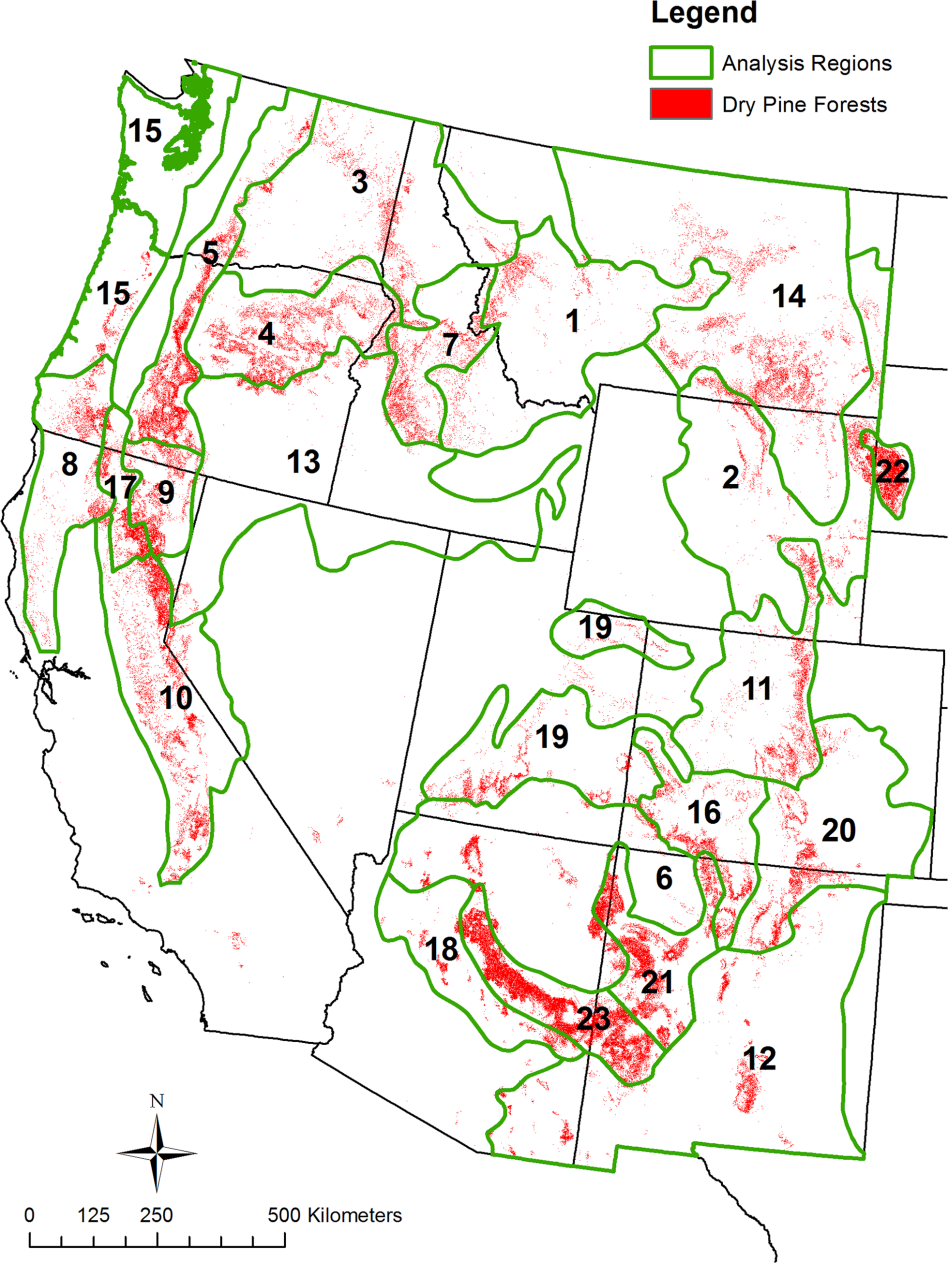
Dry pine analysis regions and dry pine forest area.

**Fig 2:**
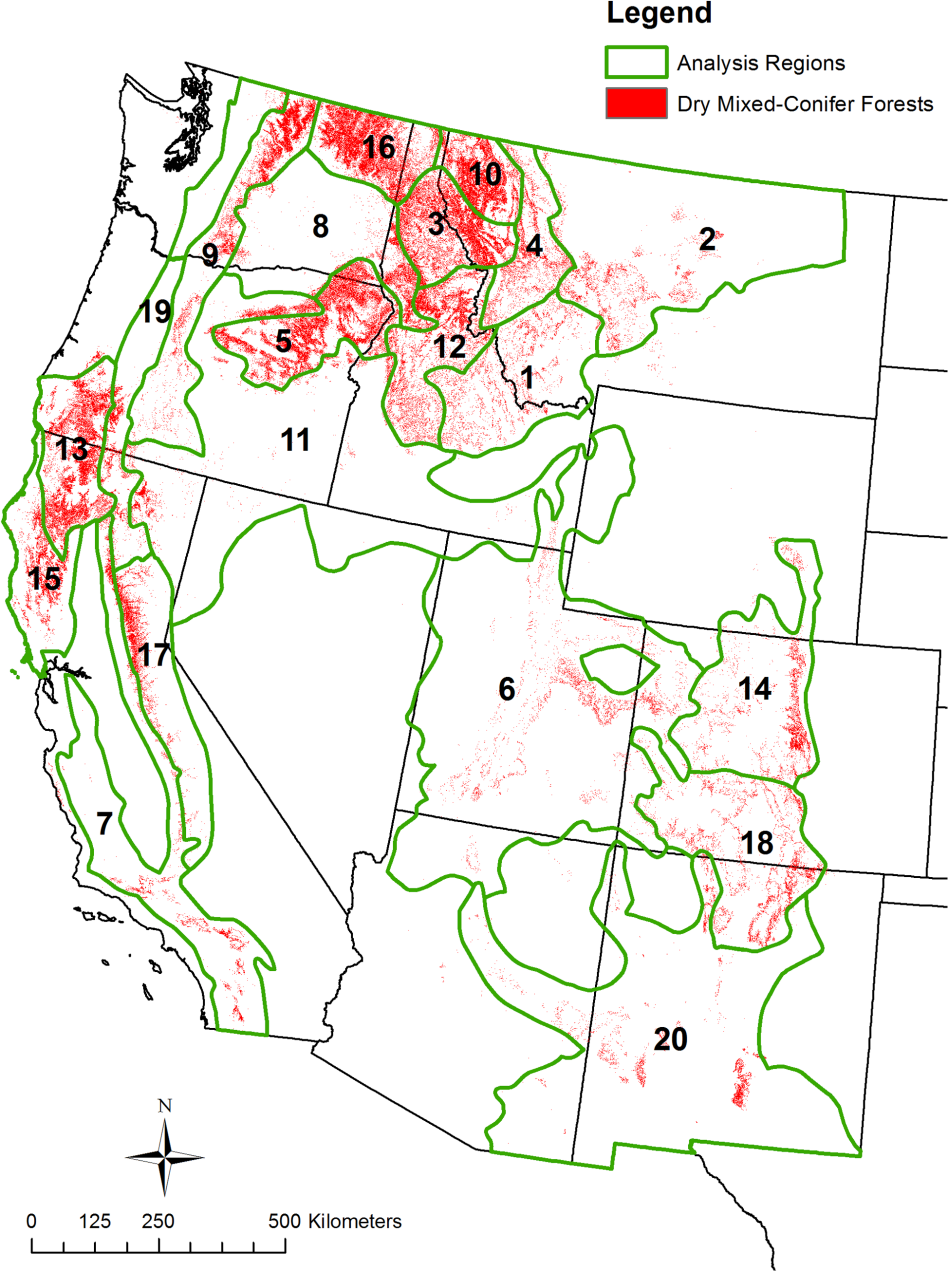
Dry mixed-conifer analysis regions and dry mixed-conifer forest area.

I thus used only two categories, not detailed Ecological Systems, for analysis: dry pine forests and dry mixed-conifer forests (Table 2). Dry pine forests cover 12.6 million ha (Fig 1, Table 3) and include nine Ecological Systems (Table 2). Although *Pinus ponderosa* often dominates, dry pine forests also include *Pinus jeffreyi* forests, which in places intermix with, and are similar to *P*. *ponderosa* forests, and Madrean pine-oak forests with a diversity of pines. Dry mixed-conifer forests cover 12.9 million ha (Fig 2, Table 4) and include eight Ecological Systems (Table 2). They have the pines with associated firs (*Abies*, *Pseudotsuga*). I omitted minor types that did not fit these categories. Total analysis area is about 25.5 million ha.

**Table 2:** Two categories of vegetation used in the analysis and their constituent Landfire biophysical settings and Ecological Systems.

Landfire Biophysical Code	Ecological System
*Dry Pine Forests*	
10310	California Montane Jeffrey Pine-(Ponderosa Pine) Woodland
10600	East Cascades Oak-Ponderosa Pine Forest and Woodland
10240	Madrean Lower Montane Pine-Oak Forest and Woodland
10300	Mediterranean California Lower Montane Black Oak-Conifer Forest & Woodland
11650	Northern Rocky Mountain Foothill Conifer Wooded Steppe
10530–10532	Northern Rocky Mountain Ponderosa Pine Woodland and Savanna
10790–10792	Northwestern Great Plains-Black Hills Ponderosa Pine Woodland and Savanna
11170–11172	Southern Rocky Mountain Ponderosa Pine Savanna
10540–10542	Southern Rocky Mountain Ponderosa Pine Woodland
*Dry Mixed-Conifer Forests*	
10210	Klamath-Siskiyou Lower Montane Serpentine Mixed Conifer Woodland
10260	Madrean Upper Montane Conifer-Oak Forest and Woodland
10270	Mediterranean California Dry-Mesic Mixed Conifer Forest and Woodland
10450	Northern Rocky Mountain Dry-Mesic Montane Mixed Conifer Forest
10451	Northern Rocky Mountain Dry-Mesic Montane Mixed Conifer Forest-Ponderosa
10452	Northern Rocky Mountain Dry-Mesic Montane Mixed Conifer Forest-Larch
10453	Northern Rocky Mountain Dry-Mesic Montane Mixed Conifer Forest-Grand fir
10510	Southern Rocky Mountain Dry-Mesic Montane Mixed Conifer Forest & Woodland

**Table 3:** Dry pine forests, high-severity fire rotations (FR), trends, and differences between recent or projected high-severity fire rotation and the range of historical high-severity fire rotations.

				Trend in area burned		Trend in fraction burned		Recent (1984–2012)	Low Projection (2046–2065)		High Projection (2046–2065)	
Analysis region	Dry pine area (ha)	High-severity area (ha)	High-severity FR (years)	z	p^1^	z	p1	Difference & Trend^2^	Fire^3^	Difference & Trend	Fire^3^	Difference & Trend
1	299,559	9,166	948	1.804	0.1509	0.254	0.5177	Too long-N	1.71	In range-N	2.69	In range-N
2	428,511	11,618	1,070	2.010	0.1112	1.585	0.1791	Too long-N	1.56	In range-N	-	-
3	414,399	3,838	3,131	1.145	0.2520	-0.338	0.5077	Too long-N	1.56	Too long-N	-	-
4	774,589	12,328	1,822	0.282	0.5109	-0.830	0.8350	Too long-N	1.71	Too long-N	2.69	In range-N
5	986,479	16,779	1,705	2.013	0.1116	0.772	0.4005	Too long-N	1.42	Too long-N	2.54	In range-N
6	446,322	6,591	1,964	2.123	0.1069	-0.117	0.6322	Too long-N	1.63	Too long-N	2.24	Too long-N
7	465,262	25,365	532	1.182	0.2520	1.626	0.1760	In range-N	1.71	In range-N	2.69	Too short-N
8	355,088	4,494	2,292	0.528	0.4537	0.654	0.4240	Too long-N	1.42	Too long-N	2.54	Too long-N
9	418,756	6,655	1,825	1.747	0.1600	0.677	0.4240	Too long-N	1.42	Too long-N	2.54	In range-N
10	968,322	59,773	470	0.807	0.3932	0.094	0.5833	In range-N	1.42	In range-N	2.54	Too short-N
11	463,235	9,834	1,366	2.594	0.0550 ^▀^	1.667	0.1690	Too long-N	1.71	In range-N	2.69	In range-N
12	392,914	23,394	487	2.687	0.0503 ^▀^	0.021	0.5931	In range-N	1.63	In range-N	2.24	In range-N
13	208,323	10,965	551	0.676	0.7962	-1.042	0.8810	In range-N	1.56	In range-N	-	-
14	749,365	36,485	596	2.987	0.0411 *	1.981	0.1116	In range-Y	1.62	In range-Y	2.00	In range-Y
15	95,560	0	-	-	-	-	-	Too long-N	-	-	-	-
16	451,768	7,216	1,816	2.332	0.0800	0.542	0.4537	Too long-N	1.71	Too long-N	2.69	In range-N
17	349,208	3,405	2,974	1.163	0.2520	-0.417	0.7469	Too long-N	1.42	Too long-N	2.54	Too long-N
18	223,819	4,519	1,436	3.300	0.0220 *	0.762	0.4005	Too long-Y	1.63	Too long-Y	2.24	In range-Y
19	330,118	4,299	2,227	1.523	0.1817	-0.677	0.7962	Too long-N	1.56	Too long-N	-	-
20	460,167	9,440	1,414	2.425	0.0704	1.420	0.2133	Too long-N	1.62	Too long-N	2.00	In range-N
21	1,101,877	2,124	15,043	1.424	0.2118	-0.613	0.7962	Too long-N	1.63	Too long-N	2.24	Too long-N
22	469,395	6,021	2,261	1.321	0.2269	1.336	0.2269	Too long-N	1.62	Too long-N	2.00	Too long-N
23	1,855,620	78,455	686	3.264	0.0220 *	1.294	0.2269	In range-Y	1.63	In range-Y	2.24	In range-Y
Total ^4^	12,603,579	349,959	1,045	2.682	0.0503 ^▀^	0.319	0.5077	Too long-N	1.59^5^	In range-N	2.41^4^	In range-N

Analysis regions are in Fig 1. All burn areas are corrected for missing small fires by dividing initial estimates by 0.95.

^1^ Trends significant at α < 0.05 are starred (*), trends that are close to significant (p < 0.06) have a dark square (^▀^). The p-values are from the Mann-Kendall trend test after the Benjamini-Hochberg correction for *n* = 88 trend tests.

^2^ Differences between recent or projected high-severity fire rotations and the range of historical high-severity fire rotations are categorized as: (1) In range, if recent or projected high-severity fire rotation was within the range of available historical estimates, (2) too short, if recent or projected high-severity fire rotation was outside and shorter than the range of historical estimates, and (3) too long, if recent or projected high-severity fire rotation was outside and longer than the range of historical estimates. “Y” indicates there was a significant upward trend in area burned at high severity, and “N” indicates there was not.

^3^ The ratio of future area burned to recent area burned from the low and high projections by Yue *et al*. [42]

^4^ The total excludes 105,077 ha of dry pine forests not in the 23 analysis regions and not included in the analysis

^5^ This is the mean across the regions for which there is a projection

**Table 4:** Dry mixed-conifer forests, high-severity fire rotations (FR), trends, and differences between recent or projected high-severity fire rotation and the range of historical high-severity fire rotations.

				Trend in area burned		Trend in fraction burned		Recent (1984–2012)	Low Projection (2046–2065)		High Projection (2046–2065)	
Analysis region	Dry mixed conifer area (ha)	High-severity area (ha)	High-severity FR (yrs)	z	p^1^	z	p^1^	Threat & Trend^2^	Fire^3^	Threat & Trend	Fire^3^	Threat & Trend
1	389,489	11,287	1,001	2.087	0.1112	-0.665	0.7962	Too long-N	1.71	In range-N	2.69	In range-N
2	341,577	9,593	1,033	0.324	0.5077	0.025	0.5931	Too long-N	1.62	In range-N	2.00	In range-N
3	1,258,666	7,130	5,119	2.002	0.1112	-0.050	0.6184	Too long-N	1.71	Too long-N	2.69	Too long-N
4	423,653	23,451	524	1.580	0.1791	-1.136	0.8891	In range-N	1.71	In range-N	2.69	Too short-N
5	1,510,344	31,397	1,395	0.302	0.5093	-1.169	0.8891	Too long-N	1.71	In range-N	2.69	In range-N
6	374,905	9,802	1,109	2.664	0.0503 ^▀^	0.415	0.4890	Too long-N	1.56	In range-N	-	-
7	298,545	40,867	212	1.144	0.2520	1.369	0.2200	Too short-N	1.24	Too short-N	-	-
8	478,717	8,334	1,666	0.667	0.4240	0.688	0.4240	Too long-N	1.56	Too long-N	-	-
9	799,591	49,730	467	2.201	0.0948	-0.257	0.6869	In range-N	1.42	In range-N	2.54	Too short-N
10	845,686	11,313	2,168	0.446	0.4811	-0.109	0.6322	Too long-N	1.71	Too long-N	2.69	In range-N
11	430,935	6,246	2,001	0.638	0.4240	-0.573	0.7962	Too long-N	1.56	Too long-N	-	-
12	909,493	70,987	372	0.844	0.3807	1.205	0.2508	In range-N	1.71	In range-N	2.56	Too short-N
13	1,204,586	48,025	727	1.678	0.1690	0.374	0.5025	In range-N	1.42	In range-N	2.54	In range-N
14	373,118	22,132	489	2.210	0.0948	0.090	0.5833	In range-N	1.71	In range-N	2.69	Too short-N
15	419,845	17,881	681	0.629	0.4240	1.552	0.1817	In range-N	1.42	In range-N	2.54	In range-N
16	1,356,500	4,974	7,909	0.191	0.5931	-1.713	0.9570	Too long-N	1.71	Too long-N	2.69	Too long-N
17	420,709	14,228	858	1.276	0.2302	0.600	0.4306	Too long-N	1.42	In range-N	2.54	In range-N
18	509,264	15,943	926	2.878	0.0440*	1.006	0.3070	Too long-Y	1.71	In range-Y	2.69	In range-Y
19	270,897	9,328	842	1.538	0.1817	1.664	0.1690	In range-N	1.42	In range-N	2.54	In range-N
20	302,471	14,823	592	2.515	0.0587 ^▀^	1.304	0.2269	In range-N	1.63	In range-N	2.24	In range-N
Total^4^	12,918,991	427,471	875	1.895	0.1276	0.506	0.4564	In range-N	1.58^5^	In range-N	2.56^5^	In range-N

Analysis regions are in Fig 2. All burn areas are corrected for missing small fires by dividing initial estimates by 0.95.

^1^ Trends significant at α < 0.05 are starred (*), trends that are close to significant (p < 0.06) have a dark square (^▀^). The p-values are from the Mann-Kendall trend test after the Benjamini-Hochberg correction for *n* = 88 trend tests.

^2^ Differences between recent or projected high-severity fire rotation and the range of historical high-severity fire rotations are categorized as: (1) In range, if recent or projected high-severity fire rotation was within the range of available historical estimates, (2) Too short, if recent or projected high-severity fire rotation was outside and shorter than the range of historical estimates, and (3) Too long, if recent or projected high-severity fire rotation was outside and longer than the range of historical estimates. “Y” indicates there was a significant upward trend in area burned at high severity, and “N” indicates there was not.

^3^ The ratio of future area burned to recent area burned from the low and high projections by Yue *et al*. [42]

^4^ The total excludes 68,530 ha of dry mixed-conifer forests not in the 20 analysis regions and not included in the analysis

^5^ This is the mean across the regions for which there is a projection

### Analysis regions and data on high-severity fire

I modified Bailey’s Ecoregions [49–50] to analyze geographical variation across dry forests. I clipped the Ecoregion map to the eleven western states, plus the Black Hills, which together contained 20 provinces and 80 sections. Provinces are based on vegetation types and finer sections are based on terrain [49]. I combined adjacent sections, with similar physiographic setting, to create “analysis regions” that are generally each > 250,000 ha (Figs 1 and 2), so they would be several times larger than expected maximum fire sizes. I could not always achieve this, as a similar adjoining section was not always available. I did this separately for dry pine forests and dry mixed-conifer forests, as their contiguous areas > 250,000 ha were not congruent.

Area-burned data are from the Monitoring Trends in Burn Severity (MTBS) program [51], a government program that compares Landsat satellite data before and after fires, supplemented by plot data, to classify and map burn severity [52]. Although fire severity may be species-dependent [21], MTBS uses a standard protocol, based on pre-fire and post-fire satellite data and both the differenced normalized burn ratio (dNBR) and a relativized version of this ratio (RdNBR), to define fire-severity classes in relation to canopy tree mortality [52]. Burn severity is mapped as background data (not part of the fire), non-mappable areas (e.g., due to clouds), increased vegetation response or greenness not likely to indicate fire, plus four burn-severity classes: 1 = unburned to low severity, 2 = low severity, 3 = moderate severity, 4 = high severity. Here, I focused on class 4, but used classes 1–3 to analyze fraction of high-severity fire. I used MTBS data for actual area burned, not just burn perimeters, as perimeters contain substantial unburned area [53]. I used MTBS national “burn-severity mosaics,” which are just maps of all fires in a particular year (30 m resolution), as it was infeasible to process data for individual fires across 25.5 million ha. The dataset is defined by the April 16, 2014 MTBS data release, which provides full coverage of the 29 years from 1984–2012. Fires are nearly all wildfires, only about 2% prescribed fires, few of which likely burned at high severity. MTBS data cover fires greater than about 405 ha, which MTBS suggests account for about 95% of total burned area [51]. I divided initial area-burned totals by 0.95 to roughly correct for missing fires < 405 ha in area.

I completed GIS analyses in ArcGIS 10.2, where I projected all maps to NAD 1983, Albers Conic Equal Area Projection, if they did not have this projection, and to rasters with 30-m resolution pixels. I first intersected the map of the two categories of forests with the map of analysis regions. Then, I calculated the area of each analysis region in each category. Next, I used the area of each category in each region individually as a “mask,” which restricts all analysis and area reporting to the mask area. Finally, I extracted each year’s MTBS map of area burned within that category, and reported area burned at high severity.

### Statistical analysis

I used the Mann-Kendall non-parametric statistic, widely used to analyze trend in non-normal time-series data [54], to test the null hypothesis of no upward trend (a one-tailed test) in area burned at high severity and fraction of total area burned that burned at high severity in each analysis region, based on α = 0.05. However, there are 88 trend tests, and the probability of finding at least one to be significant by chance is high, thus the tests must be corrected to avoid false positives from multiple tests. The false positive is that a trend is found that did not exist, and the false negative is that a trend is not found that did exist. Bonferroni correction reduces false positives, but increases false negatives, thus I used Benjamini-Hochberg correction, with the p.adjust program in R, to control false positives for the 88 trend tests and also control false negatives [55]. Unlike Bonferroni correction, which adjusts alpha, this method corrects the p-value for the chosen alpha. Prior to completing the trend analyses I tested the null hypothesis of no temporal autocorrelation (α = 0.05), for up to 7-year lags, in the 29-year time series for each of the 88 cases using Minitab 16.0. None of the series had significant autocorrelation after Benjamini-Hochberg correction of p-values from the Ljung-Box Q test statistic.

I did two other tests to gain further understanding of fraction burned at high severity. First, I compared, using two one-sample *t*-tests, recent mean fraction of high-severity fire to mean fractions of high severity from GLO and early aerial-photo reconstructions (Table 1), which showed the percentage of each historical landscape with evidence of low-, mixed- and high-severity fire evident in the late-1800s from fires that burned in the preceding 100–140 years [28]. These tested whether the recent fraction of high-severity fire was historically unprecedented, was near, or was deficient relative to historical fractions. For dry pine, there is one estimate each from eastern Oregon [29], the Mogollon Plateau [28] and the Coconino Plateau [56], which are mostly dry pine. For dry mixed conifer, there is one from eastern Oregon [29], two from the western Sierra Nevada [30], three from Colorado, Arizona, and Oregon [28], and one from eastern Washington [26]. These have some limited dry pine. I also compared, using a two-sample *t*-test, recent mean fractions of total area burned that burned at high severity, across the 29-year period, between dry pine and dry mixed-conifer forests. This clarified whether more high-severity fire was occurring recently in dry mixed-conifer than dry pine forests.

### Comparing fire rotations in recent and historical periods

To calculate recent high-severity fire rotation for each analysis region, I summed area burned at high severity across the 29 years for each forest type in each region. I used the formula for fire rotation [21]: Period of analysis / fraction of analysis region burned. Fraction of area burned is area burned (ha) at high severity in the category and region over the 29-year period / total area in the category in the analysis region (Tables 3 and 4).

To evaluate recent rates of high-severity fire relative to historical rates, I compared recent high-severity fire rotations to historical rotations. I compiled reconstructions of historical high-severity fire rotations in dry forests in the study area (Table 1). These provided insufficient data for region-by-region comparison across the study area. Thus, as a first approximation, I used the range of available estimates from GLO data, FIA data, and aerial photographs, which is 217–849 years (Table 1), as the standard to compare with recent rates in each region. Estimates from charcoal in sediments are provided only as corroborative evidence for historical rates (Table 1). Charcoal study sites may not be random samples, but likely were selected without bias.

I did not include studies of fire severity from early timber inventories [57–62] in Table 1, because these inventories have too many limitations. First, timber inventories were done in areas unrepresentative of larger landscapes [58], primarily where timber sales were likely [63] and inventory tree data are unrepresentative even within these inventory areas. This is because tree data were usually only recorded for merchantable forests that typically had large trees at low density associated with low-severity fires; data were often not recorded for younger, denser forests or recovering burned areas within an overall inventory area [62, 63]. For example, about 70% of one inventory area had no recorded tree data [58]. Recent authors [57–61] did not adjust estimates for these unsampled areas, although adjustments were standard inventory protocol before 1917 [63]. These published data [57–61] thus do not provide valid statistical samples of even the overall inventory area, much less the larger surrounding landscape. Second, inventories typically included written records of fire severity for the section within which inventory transects occurred [62], including reports of severe fires, but these key records were not used in past studies [57–61]. When these fire-severity records were found and included in a new study, abundant high-severity fire was found, with estimated high-severity fire rotations congruent with earlier GLO reconstructions over larger areas [62]. Finally, by 1910, understory fuels and small trees had been reduced by livestock grazing, early miners and herders had set fires, and logging and other human disturbances had altered forests [64]. Thus, data from early timber inventories [57–61] are not valid samples of historical forests and studies that omit or lack records of high-severity fire [57–61] do not provide valid estimates of rates and patterns of high-severity fire.

GLO-based reconstructions of fire severity, the main historical source of rates of high-severity fire (Table 1), have been calibrated, validated, and corroborated [27–29, 40, 56, 65], but some critiques missed this testing. Fulé et al. [66] suggested structure-based models we used to reconstruct fire severity were not validated or corroborated. However, they missed our methods section that explains how we calibrated and validated our models with tree-ring reconstructions [28] and they missed a summary of evidence corroborating our findings with independent reconstructions and multiple lines of evidence [28, 65]. We added further corroboration of our reconstructions in our reply [65], subsequent studies [29–30, 40, 56], and here (Table 1). Another example of missing our testing is from Maxwell et al. [67] and Collins et al. [58], who said our fire-severity reconstructions used data from bearing trees selected by surveyors in a biased manner. Neither set of authors cited our study of surveyor bias and error, done specifically in the dry forests where our GLO reconstructions were done, in which we found low levels of bias and error [68]. Finally, several recent studies, using the early timber inventories, discussed above, in areas partially overlapping or near our GLO-reconstruction areas, found low tree densities and little or no high-severity fire, and suggested this shows that our GLO reconstructions were in error [57–61]. However, as discussed above, it is timber-inventory studies that omitted fire-severity records [57–61], and that were not validated or corroborated, that likely are in error.

Using the best available data on historical rates of high-severity fire, which included estimates from GLO reconstructions, analysis of early aerial photography, and FIA data (Table 1), I classified recent high-severity fire rotations, relative to the range of historical fire rotations, which is 217–849 years (Table 1). The classes are: (1) within range, if the recent high-severity fire rotation was within the historical range, (2) too short, if the recent high-severity fire rotation was outside and shorter than the historical range, and (3) too long, if the recent high-severity fire rotation was outside and longer than the historical range.

To evaluate future projected increases in high-severity fire, I compared projected high-severity fire rotations to the historical high-severity fire rotations estimated by GLO, aerial photo, and FIA data, and classified the outcomes as above (e.g., too long). To estimate future high-severity fire rotations, I separately applied both the low and high regional projections of Yue *et al*. [42] reported in the introduction. I used the Yue *et al*. projections, as others do not report expected changes in area burned or do not provide detail for the western United States (e.g., [69–71]). To use Yue *et al*. by region, I divided recent high-severity fire rotations by the projected ratio of future area burned to recent area burned at any severity after cross-walking ecoregions in Yue *et al*. with my analysis regions. I show later there was no statistically significant upward trend in fraction of fire that burned at high severity recently, thus I assumed future fire will not have an increased fraction of high-severity fire.

## Results

### Recent versus historical fire rotations

**Fig 3:**
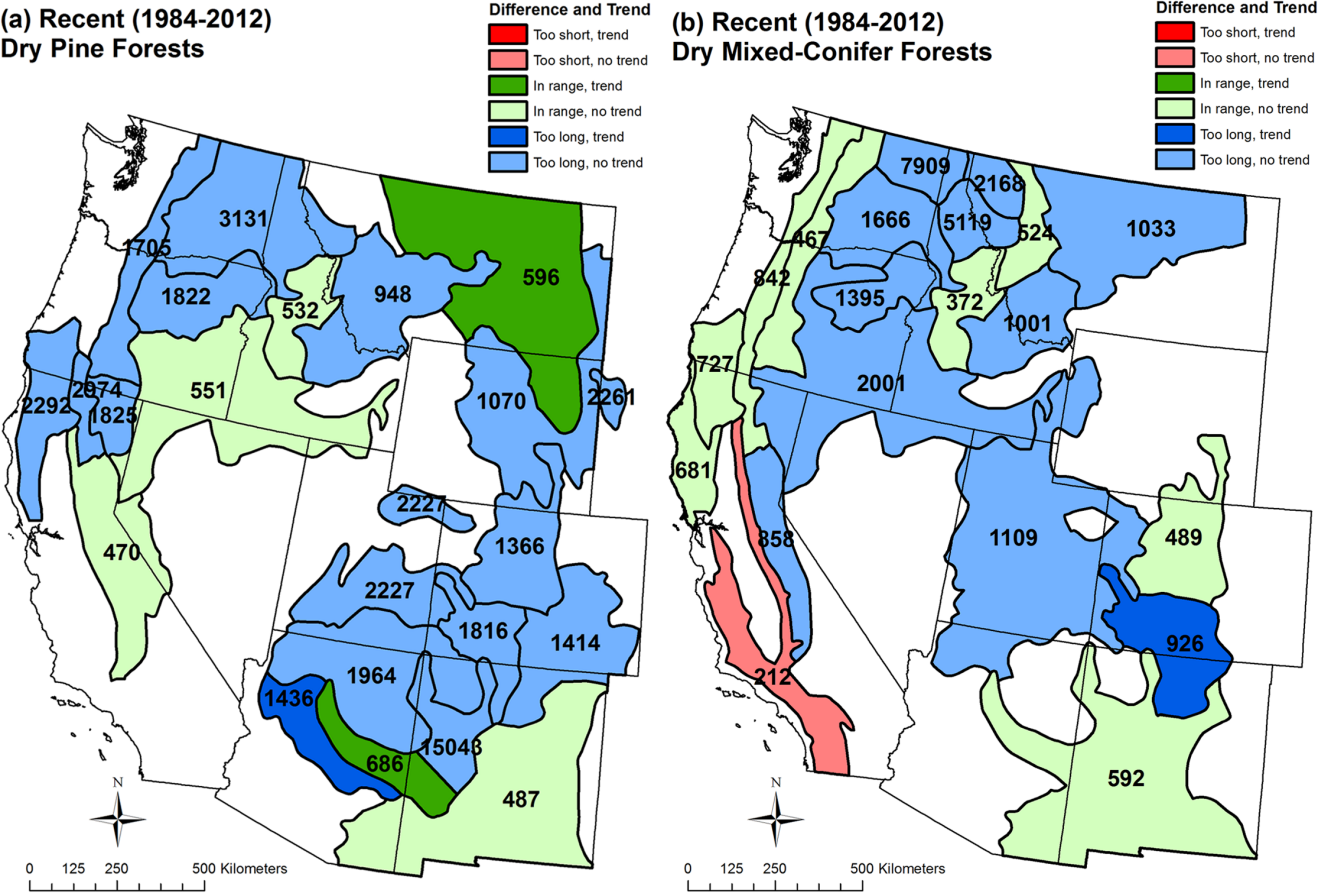
Differences between recent (A.D. 1984–2012) high-severity fire rotation and historical range of high-severity fire rotations, recent trends, and recent fire rotations for high-severity fire in (a) dry pine forests and (b) dry mixed-conifer forests by analysis region. High-severity fire rotation (years), from Tables 3 and 4, is printed over each region, and represents the expected time to burn, at high severity, an area equal to the region. Colors correspond with data in Table 3 for dry pine and Table 4 for dry mixed conifer forests. Differences are: (1) “In range,” if recent high-severity fire rotation was within the range of historical estimates, (2) “Too short,” if outside and shorter than historical estimates, and (3) “Too long,” if outside and longer than historical estimates. “Trend” indicates that a statistically significant upward trend in area burned at high severity was found in a region, and “No trend” indicates one was not found, with data shown in Tables 3 and 4. Regions that lacked a statistically significant upward trend in area burned at high severity (Tables 3, 4) have lighter shading. Several Bailey sections were merged or split to create the analysis regions.

High-severity fire rotations from 1984–2012 were within the historical range or were too long, relative to historical high-severity fire rotations, overall across the western USA in both dry pine and mixed-conifer forests and individually in 41 of 42 analysis regions (Fig 3, Tables 3 and 4). Fire rotation was not calculated in Region 15 in dry pine, as no high-severity fire occurred (Table 3). Overall, recent high-severity fire rotations of 1,045 years in dry pine and 875 years in dry mixed-conifer forests were a little too long, relative to the range of historical fire rotations of 217–849 years, meaning too little recent high-severity fire relative to historical high-severity fire (Tables 1, 3 and 4). In dry pine forests, recent high-severity fire rotations were within the range of historical fire rotations in 6 of 22 regions and were too long, meaning a deficiency of high-severity fire, in 16 of 22 regions (Fig 3A, Table 3). In dry mixed-conifer forests, recent high-severity fire rotations were within the range of historical rotations in 8 of 20 regions, were too long in 11 of 20 regions, but too short, producing too much high-severity fire relative to historical fire, in one region in southern California (Fig 3B, Table 4).

### Recent trends

**Fig 4:**
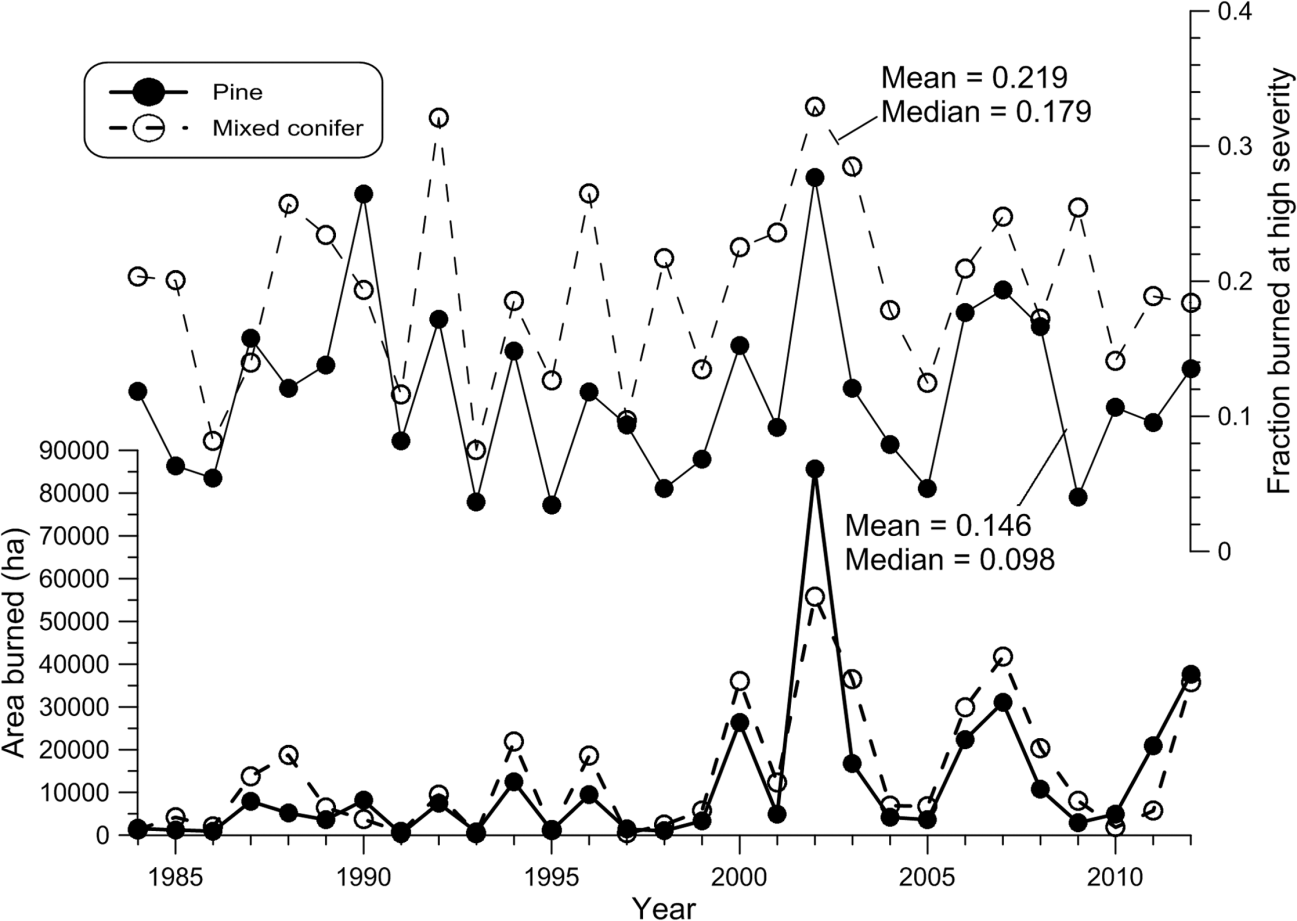
Trends between 1984–2012 in area burned at high severity (bottom) and fraction burned at high severity (top). Results are shown for both dry pine and dry mixed conifer forests.

Statistically significant upward trends were lacking overall for area burned at high severity from 1984–2012 in both dry pine (*z* = 2.682, *p* = 0.0503) and in dry mixed-conifer forests (*z* = 1.895, *p* = 0.1276). The dry-pine trend was very close to significant. Across analysis regions, statistically significant upward trends in area burned were found in only 3 of 23 dry pine regions (Fig 3A, Table 3) and1 of 20 dry mixed-conifer regions (Fig 3B, Table 4) in parts of the Southwest and Rocky Mountains. Two regions were close to significant in dry pine and two in dry mixed conifer, also in the Southwest and Rocky Mountains (Tables 3 and 4). Interannual fluctuations in area burned closely matched in dry pine and mixed-conifer forests (*r* = 0.856, *p* < 0.001), and area burned was concentrated in known major fire years (A.D. 1994, 1996, 2000, 2002, 2006–2007, 2011–2012), particularly since A.D. 2000 (Fig 4).

Statistically significant upward trends were also lacking overall for fraction of high-severity fire from 1984–2012 in both dry pine (*z* = 0.319, *p* = 0.508) and in dry mixed conifer forests (*z* = 0.506, *p* = 0.456) (Fig 4, Tables 3 and 4). Statistically significant upward trends in fraction burned at high severity were also lacking in all 23 dry-pine regions and all 20 dry mixed-conifer regions (Tables 3 and 4). Interannual fluctuations in fraction burned at high severity also significantly matched in dry pine and mixed-conifer forests, but not as closely (*r* = 0.520, *p* = 0.004) as for area burned (Fig 4). Fraction burned at high severity was higher in major fire years, as was area burned, but not more so since A.D. 2000 (Fig 4).

For dry pine, the recent mean fraction of high-severity fire for the whole study area (0.117) was not significantly (α = 0.05) different from the mean (0.111) of historical reconstructions (*t*(2) = -0.15, *p* = 0.897). However, for dry mixed conifer, the recent mean fraction for the whole study area (0.194) was significantly lower than the mean (0.356) of the seven reconstructions (*t*(6) = 6.18, *p* = 0.001). Thus, these comparisons suggest that the recent mean fraction of high-severity fire is not unprecedented, but instead congruent with historical mean fraction of high-severity fire in dry pine and too low relative to historical mean fraction of high-severity fire in dry mixed conifer.

A *t*-test showed a significant difference, in mean fraction of high-severity fire across the 29 years in the analysis period (Fig 4), between dry pine (Mean = 0.117, s.d. = 0.063) and mixed-conifer forests (Mean = 0.194, s.d. = 0.070); *t* (55) = -4.51, *p* < 0.001. This shows that a higher fraction of high-severity fire (1.66 times) occurred recently in mixed conifer as in pine.

### Projections

**Fig 5:**
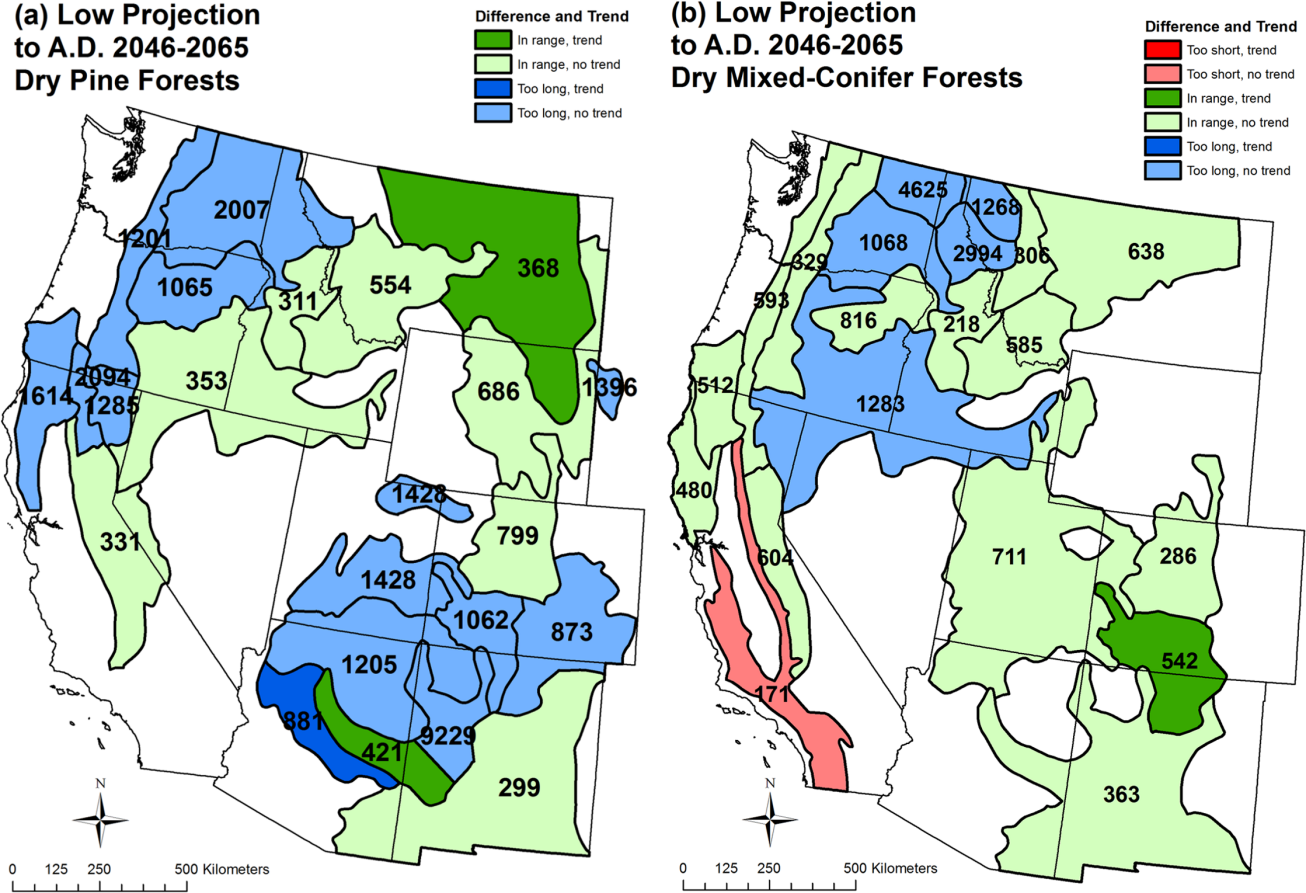
Low projection (to A.D. 2046–2065) of differences relative to the historical range of high-severity fire rotations, given 1984–2012 trends, and projected fire rotations for high-severity fire in (a) dry pine forests and (b) dry mixed-conifer forests by analysis region. See Fig 3 for an explanation of figure contents.

Low projections [42] in dry-pine regions for 2046–2065 would be restorative of the high-severity fire process or provide ongoing maintenance of high-severity fire at historical rates, with no region having a fire rotation too short relative to historical high-severity fire rotations (Fig 5A, Table 3). Of 22 dry-pine regions (region 15 excluded since no high-severity fire recently), 13 that had high-severity fire rotations that were too long up to 2012 still had rotations too long by 2046–2065, but were closer to historical high-severity rotations, thus partially restored, while 3 of the 22 regions changed from too long to within range, indicating restoration of high-severity fire to historical rates. The remaining six regions that were within range by 2012 stayed in this category, thus had ongoing maintenance of high-severity fire at historical rates.

Low projections in dry mixed-conifer regions would also be restorative or provide ongoing maintenance of high-severity fire at historical rates, except in one region (Fig 5B, Table 4). Five of 20 dry mixed-conifer regions with high-severity fire rotations that were too long up to 2012 would still have fire rotations too long, but would be closer to historical rates, thus would have high-severity fire rates partially restored. Six of 20 mixed-conifer regions with high-severity fire rotations that were too long up to 2012 would change to within range, indicating restoration of high-severity fire to historical rates. Eight of 20 mixed-conifer regions with high-severity fire rates within range in 2012 would remain within range, indicating maintenance of high-severity fire at historical rates. One region, however, that already had a too short high-severity fire rotation by 2012, was projected to have an even shorter high-severity fire rotation.

**Fig 6:**
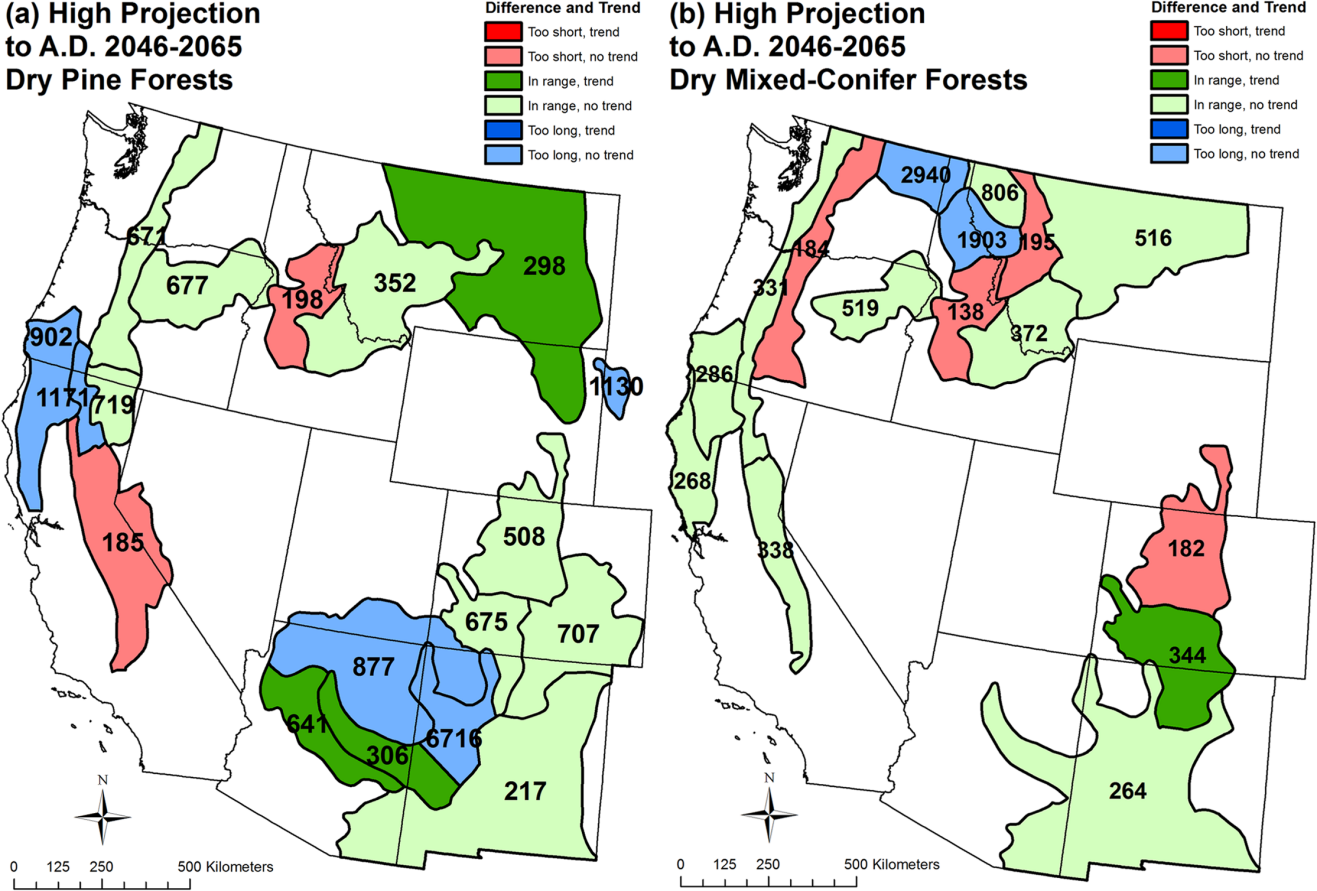
High projection (to A.D. 2046–2065) of differences relative to the historical range of high-severity fire rotations, given 1984–2012 trends, and projected fire rotations for high-severity fire in (a) dry pine forests and (b) dry mixed-conifer forests by analysis region. Several analysis regions are omitted, because the high projections by Yue *et al*. [42] were not possible for those areas. See Fig 3 for an explanation of figure contents.

High projections in dry-pine regions still would be predominantly restorative of high-severity fire or provide ongoing maintenance of high-severity fire at historical rates in 15 regions, but two regions would have high-severity fire rotations too short, relative to historical high-severity fire rotations (Fig 6A, Table 3). High projections in dry mixed-conifer regions would also be predominantly restorative of historical high-severity fire rates or provide ongoing maintenance of high-severity fire at historical rates in 12 regions, but four regions would have fire rotations too short relative to historical high-severity fire rotations (Fig 6B, Table 4). High projections are missing for four of 22 pine regions and four of 20 mixed-conifer regions (Fig 6, Tables 3 and 4), because fire-climate relationships were not found that allowed projections [42].

## Discussion

Comparison of recent and historical high-severity fire rotations shows that high-severity fire is not occurring in dry forests at rates that are exceptionally high relative to the range of historical rates. Recent high-severity fire instead is deficient (too long) overall across dry pine and dry mixed-conifer forests relative to historical rates. Recent high-severity fire rotations are quite long, averaging 1,045 years in dry pine and 875 years in dry mixed conifer, more than ample time for forests to recover and reach very old age before the next high-severity fire. High-severity fire rotations in dry forests are also within the historical range or are too long relative to historical fire rotations in all but one of 43 regions of the western USA up to A.D. 2012. Absence of significant overall recent upward trends in area burned and in fraction burned at high severity also suggests high-severity fire is not significantly increasing or becoming more severe. The trend is quite close to significance for area burned in dry pine forests, which I discuss below. These findings do not support the hypothesis that high-severity fire is occurring at exceptionally high rates in dry forests or is generally increasing or becoming more severe [6–7].

The location of the few regions (4 of 43) with a statistically significant upward trend in area burned at high severity over the 1984–2012 period, in parts of the Southwest and Rocky Mountains, is consistent with previous studies in broader forest types [3–4]. This consistency suggests stronger climatic than fuel influences [2–4]. A strong climatic role in upward trends is also supported by the high correlation of interannual fluctuations in area burned at high severity between dry pine and dry mixed conifer and by concentration of area burned at high severity in major fire years. However, part of the explanation of trends in these particular regions and the nearly significant trend overall in dry pine forests may not be climate, but human-set fires. Two of the dry pine regions with statistically significant upward trends, in central Arizona (Fig 3A), experienced very large human-set fires, the 2002 Rodeo-Chediski fire and the 2011 Wallow fire. In contrast, the upward trend in dry pine forests in eastern Montana is likely not related to human-set fires, but instead to three exceptional lightning-ignited fires (Ash Creek, Rosebud Creek, Chalky) in 2012 as well as earlier natural fires. Disentangling the contributions of human-set fires and climatic change to trends is beyond the scope of this study, but is needed.

Projections to 2046–2065 suggest high-severity fire would be predominantly restorative of the high-severity fire process or provide ongoing maintenance of high-severity fire at historical rates, except in a few regions. Low projections indicate fire rotations too short only in one region of 42 total, and that region already had a fire rotation too short by 2012. Thus, dry forests generally have the capacity to absorb up to 1.71 times as much high-severity fire (the maximum increase in the low projections), beyond what was occurring up to 2012, without exceeding historical rates. The six regions, under the high projections, that would have fire rotations too short by 2046–2065, would not be able to absorb 2.54–2.69 times as much high-severity fire as in 1984–2012, but 19 other regions that received this level of increase were not pushed beyond historical rates. This suggests most dry-forest landscapes have the capacity to absorb substantial increased high-severity fire, but if high-severity fire increases above about 2 1/2 to 3 times as much as in 1984–2012, that capacity will begin to be reached. Thus, it may be at least several decades before dry-forest landscapes may generally begin to be affected by exceptional high-severity fire rates because of projected climatic change. Projections based on Yue *et al*. [42] could also be tempered by lack of actual trend in regions up to 2012, which is 35–46% of the duration from 1984 to 2046–2065. Perhaps the projection will not fully emerge and the high projections, with the greatest increase in future fire, may be less concerning. Of course, lack of recent trend does not clearly mean the Yue *et al*. [42] projections will not eventually occur.

If high-severity fire rotations that are too short do begin to appear, entire landscapes will not be affected at once, but instead effects will be lagged and heterogeneous, likely requiring centuries after onset to fully affect dry-forest landscapes. Long delays occur because fire effects accrue over time from separate ignition and spread events. If a historical fire rotation of 400 years experiences a doubling of annual area burned, and the new rotation is 200 years, then on average it will be 200 years before landscapes are fully affected by this change [72]. Lagged responses of landscapes, to changes in fire, affect both increased fire that is restorative or that leads to too-short fire rotations. The heterogeneity and lagged effects of fire contrast with those of droughts and insect outbreaks, which can alter large land areas nearly synchronously without the long delays inherent with fire. This is supported by a study that showed that major projected changes in vegetation in western North America with global warming occur mainly from direct climate effects (e.g., drought), with < 1% from wildfire [73].

### Limitations

These trend analyses and fire rotations have some inherent limitations. The Landfire Biophysical Settings maps aim to predict historical vegetation predating the trend analyses, but could still exclude non-forested area that was burned in dry forests at high-severity early in the 1984–2012 period, leading to false upward trends [17]. Fully reliable trend analysis across large land areas requires a nationally consistent and detailed vegetation map based on imagery that predates MTBS data coverage in 1984. Because I cannot definitely exclude a possible false upward trend, the four significant upward trends, and other trends close to significant, are particularly clouded by this uncertainty.

The MTBS program has provided a remarkable dataset, but 29 years is still a limitation. Estimated fire rotations are hundreds of years, and nearly a full fire rotation of data is needed to accurately estimate the rotation [72]. Trends over the 29-year period hinge on the timing and magnitude of only a few major fire peaks (Fig 4). Thus, estimated rotations are valid for the 29-year period, but subject to change as more data accrue. Data from 25.7 million ha temper this concern, but fire can be synchronized over large land areas by teleconnections with periods > 29 years [74], thus 29 years are also insufficient from the standpoint of potential climate cycles. Also, high-severity fire rotations may not be homogeneous across landscapes; high-severity fire could be favored in certain biophysical settings and dis-favored in others (e.g., 5, 39). Thus, recent fire-rotation estimates in this study represent averages for analysis regions that warrant use with caution in smaller subareas of these regions.

MTBS data, which are for fires generally > 400 ha in area, may or may not contain 95% of total burned area, as estimated by the MTBS program. One estimate for part of the Sierra Nevada was 92.8% [17]. If the actual percentage was not 95%, then fire rotations could be somewhat too low or too high. Some caution is thus warranted from this standpoint in the use of estimates, although this concern is buffered by the use of a large historical range for comparison.

The GLO-based reconstructions may be one of the few available ways to reconstruct historical severe fires in the spatially extensive manner that is needed to provide data about fire rotation, patch size, and other attributes. These reconstructions are calibrated, validated, and corroborated [27–29, 40, 56, 65], but would benefit from additional calibration and validation to improve the linkage of forest structure with fire severity and to help estimate the precision of estimates of fire rotation. Precision is not known very well, except that there is calibration and validation with tree-ring reconstructions, there is corroboration by early historical records and maps, and there is congruence between the findings of GLO-based, aerial-photo-based, and paleo-fire based methods (Table 1). GLO-based methods are typically based on 100–140 year periods before the surveys, thus less than a full historical fire rotation. These methods also cannot provide fine detail about high-severity fire patterns, since they are based on data pooled across 259–1,036-ha areas. It would be beneficial to combine GLO-based methods with tree-ring reconstructions and compare paleo-fire and GLO-based reconstructions in the same areas. More landscape-scale fire history is needed and may lead to further refinements in understanding of rates and patterns of historical high-severity fire.

The projections are first approximations. The Yue *et al*. [42] projections are not specific to dry forests or high-severity fires, are not based on dynamic vegetation models, and use only a moderate emissions scenario. Potential vegetation changes may be large [73], particularly for *P*. *ponderosa* in the Rocky Mountains and Southwest [75]. Although the projections here may appear simplistic, the Yue *et al*. projections are sophisticated. It is just that their direct transfer here to dry forests provides only first approximations and context for thinking about future fire in dry forests until projections specific to dry forests appear. The projections also assume similar continuing fire management and no non-linear responses. For example, a landscape trap [76] could arise if high-severity fire created more fire-prone landscapes that then burn increasingly at high severity, possibly increasing area burned at high severity beyond projections.

### Management issues

The evidence presented here shows that efforts to generally lower fire severity in dry forests for ecological restoration are not supported. Reducing fire severity in dry forests is a goal of the 2003 Healthy Forests Restoration Act (HFRA), the Collaborative Forest Landscape Restoration Program (CFLRP) of the 2009 Omnibus Public Land Management Act, and other government policies and programs. These laws, policies, and programs were developed before sufficient quantitative analysis of rates of recent high-severity fire was available, and with very limited information about rates of historical high-severity fire. Historical evidence, now available from multiple sources across large land areas (Table 1), combined with comprehensive recent fire-severity data, together show that high-severity fire is generally operating at or below historical rates. Thus, reducing fire-severity is fire suppression rather than restoration, as was commonly thought before these new data and analysis were available. Fire suppression is incompatible with laws and programs that mandate or encourage restoration of historical fire regimes and forest structure (e.g., Collaborative Forest Landscape Restoration Program).

In dry forests, suppressing high-severity fire that is operating at or below historical rates also has adverse ecological impacts. These adverse impacts include: (1) declining and potentially threatened native animals dependent on severely burned patches [77–78], (2) loss of biologically diverse early-successional habitat [79–80], reduction in fire-stimulated native shrubs and trees that were historically abundant [30, 64], and simplification of the landscape heterogeneity that is key to landscape resilience to future climate-change effects [81]. High-severity fires, for example, produce patches of younger forest that are less vulnerable to mortality in insect outbreaks and droughts [82]. Removing or reducing small trees in dry forests to reduce fire severity may significantly reduce the resilience of these forests to insect outbreaks and droughts by reducing advance recruitment that enables recovery [82].

Some potentially important ecological concerns have been expressed about high-severity fires even though these fires are not generally occurring at exceptional rates. First, many scientists worry that the size of high-severity fire patches in dry forests is currently exceptional relative to historical patch sizes, possibly hampering post-fire tree recruitment and other processes (e.g., 6). However, large comparisons of historical and modern patch sizes in dry forests, from GLO-based reconstructions in the Colorado Front Range [40] and western Sierra [30] show that high-severity patch sizes in both historical and modern fires ranged up to 8,000–9,400 ha, and recent and historical patch-size distributions had only minor differences.

Second, increasing drought and higher temperatures can alter the ecological effects of high-severity fires and modify recovery processes after these fires. Higher temperatures and drought stress may increase tree mortality after fire, effectively increasing fire severity, independent of fire intensity [83], although here I show that these forests have the capacity to absorb some increased fire severity. Historical and recent high-severity fire rotations are generally long enough to allow post-fire tree recruitment and natural recovery of dry forests between fires, but recruitment that may have historically been poor and episodic [84], could possibly worsen because of increased drought and high temperatures, so that forests have more difficulty recovering after fires [85].

Finally, local analysis might uncover ecologically valuable sites where high-severity fire has recently occurred at apparently exceptional rates [86–87], even though it is not in the larger region. For example, in the Big Oak Flats area of Yosemite National Park, tree-ring [87] and GLO reconstructions [30] both showed low- to mixed-severity fire occurred historically, with no high-severity fire over the preceding few hundred years, and reconstructions also agreed on low historical tree densities [30], yet the 2013 Rim Fire burned 24% of tree-ring plots at high severity [87]. Harris and Taylor viewed the 24% high-severity component of the Rim fire in Big Oak Flats as historically anomalous due to buildup of fuel from fire exclusion [87]. However, high-severity fire is fully expected after more than 400 years of predominantly low- to mixed-severity fire, given the historical high-severity fire rotation of 412 years reconstructed for southern Sierran mixed conifer [30]. High-severity fires in dry forests with moderate to long historical rotations are always locally surprising, occurring suddenly after centuries of stability [14]. Other studies have suggested old dry forests with long periods of low-severity fire are expected to suddenly burn at high severity [26]. Better evidence of concern in the Sierran mixed conifer forest is the recent high-severity fire rotation of 212 years in the analysis region (Fig 3B), but this region is dominated by more coastal forests where human-set fires may explain this fire rotation. A study specifically in the lower montane of the western Sierra found a recent fire rotation of 461 years [17], somewhat longer than the 412 year historical rotation, suggesting high-severity fire is not occurring at historically unprecedented rates, but needs updating to include the 2013 Rim fire and other recent fires. Regional scales, not the Big Oak Flat scale, are the scales that are essential to detect significant departures from historical rates of high-severity fires [14]. Nonetheless, high local ecological values, such as special species [86] could trump regional trends and warrant specific local management actions.

High-severity fires are infrequent powerful events, not unlike volcanic eruptions, tornadoes, or large floods that are almost beyond control or management. Yet, the ecological importance of large, infrequent, and often severe natural disturbances in structuring historical landscapes and maintaining their biological diversity is well established (e.g., [21, 77–80, 88–89]). Increasing appreciation of the role of large, seemingly destructive floods in maintaining the geomorphology and habitat diversity of rivers, suggests people may decide to accept and even restore infrequent large, severe natural disturbances in ecosystems for ecological reasons [90]. The best approach for high-severity fire is likely through wildland fire use [11], which is now wildland fire for resource benefit, managed by professional fire personnel, with restoration and maintenance of high-severity fire within historical rates and patterns as a goal. Land managers and the public can reduce fire risk near housing, infrastructure, and valuable resources to maximize area available for wildland fire use across adjoining lands. Communities can also facilitate wildland fire use by adopting growth boundaries to limit community expansion into dry forests and by rearranging land uses to favor fire-resistant land uses on their outskirts [21].

Recent rates of high-severity fire, although they are not generally exceeding historical rates, are leading to some intense fires burning into communities and damaging infrastructure. Projected increases in severe fire from climate change shown here could become even more serious for people. Fortunately, the inherently lagged response of landscapes to changes in fire does typically allow some time to prepare further. Based on the findings of this study, the public should be informed that infrequent high-severity fires in dry forests are not a consequence of poor forest management over the last century that can be fixed. Dry forests were historically renewed, and will continue to be renewed, by sudden, dramatic, high-intensity fires after centuries of stability and lower-intensity fires. Living in or near dry forests is inherently very dangerous, not unlike living beside a volcano or on a fault line with earthquakes. The people-fire problem is complex [21, 91–92], but if expansion of housing and infrastructure into dangerous dry forests [21, 93] were redirected into safer settings, both people and nature would benefit.
