# Correction: Are High-Severity Fires Burning at Much Higher Rates Recently than Historically in Dry-Forest Landscapes of the Western USA?

**DOI:** 10.1371/journal.pone.0141936

**Published:** 2015-12-01

**Authors:** William L. Baker

The phrase “FIA data” incorrectly appears several times in the paper and in the legend for Table 1. The correct phrase is “early historical data.” Please see the corrected Table 1 below.

**Table 1:** Reconstructions of fire rotation (FR) for high-severity fire in historical dry forests of the western USA, with corroborating evidence from sedimentary charcoal studies.

Author(s)	Location	Method^1^	High-severity FR (years) and severe fire-episode intervals2
DRY PINE FORESTS			
Baker [29]	E. Cascades, E Oregon	GLO tree data	***705***
DRY MIXED-CONIFER FORESTS			
Baker [30]	W. Sierra Nevada Mts., W California	GLO tree data and line data	***281–354***
Long *et al*. [31]	E. Cascades, E Oregon	Charcoal in sediment deposits	333^2^ ^,^ ^3^
Odion *et al*. [27]	N. Sierra Nevada Mts., W California	Early historical	***488***
Baker [29]	E. Cascades, E Oregon	GLO tree data	***496***
Fitch [32]	Jemez Mts., N New Mexico	Charcoal in sediment deposits	500? (400–667)^2^ ^,^ ^4^
COMBINED DRY PINE AND MIXED-CONIFER FORESTS			
Pierce and Meyer, [33] and Pierce *et al*., [34]	Central Idaho	Charcoal in sediment deposits	(154–286)^2^ ^,^ ^5^
Williams and Baker, [28]	Black Mesa, N Arizona	GLO tree data	***217***
Jenkins *et al*., [35]	Mogollon Plateau, N Arizona	Charcoal in sediment deposits	250 (200–400)^2^
Williams and Baker, [28]	Front Range, E Colorado	GLO tree data	***271***
Odion *et al*., [27]	E Cascades, E Washington	Aerial photos	***379–505***
Baker, [29]	E Cascades, E Oregon	GLO tree data	***435***
Bigio, [36]	San Juan Mts., SW Colorado	Charcoal in sediment deposits	> 471 (> 667)^2^ ^,^ ^6^
Colombaroli and Gavin, [37]	Siskiyou Mts, SW Oregon	Charcoal in sediment deposits	500 (142)^2^
Frechette and Meyer, [38]	Sacramento Mts., SE New Mexico	Charcoal in sediment deposits	500 (667)^2^ ^,^ ^7^
Williams and Baker, [28]	Mogollon Plateau and Black Mesa, N Arizona combined	GLO tree data	***522***
Williams and Baker, [28]	Mogollon Plateau, N Arizona	GLO tree data	***828***
Williams and Baker, [28]	Blue Mts., NE Oregon	GLO tree data	***849***

Studies are arranged by length of the fire rotation. Estimates from GLO data, early historical data, and early aerial photographs are shown in bold italics to emphasize their higher precision, while corroborative, less certain estimates from charcoal records are shown in regular type. The range of estimates in bold is used as the reference in this study.

^1^ Methods for reconstruction included using charcoal data from sediment, using early aerial photographs or historical records, using the GLO tree data and a calibrated model [28]. I did not use the GLO line data’s direct records of entry and exit in burned areas, as these records represent moderate- to high-severity fires, not exclusively high-severity fires [40].

^2^ These are intervals between severe fire episodes evident in alluvial deposits, that could approximate high-severity fire rotations, but are uncertain since area burned is not known and fire severity is more approximately reconstructed than with other methods. I considered data for the last 500 years from each paleo-environmental study, but also included in parentheses the interval between episodes in the last 2000 years, where this is available.

^3^ These authors indicate that it is difficult to determine fire severity from their methods, and only identify the recent fire frequency as 3 per 1000 years, but they indicate that the documented fire episodes were followed by up to 100 years of recovery, which does suggest severe fires, although this is my interpretation.

^4^ Fitch [32] suggested that low-severity fire dominated from 870 cal yr BP, but explains the possibility, but uncertainty, of a severe fire around 400 cal yr BP (p. 40), thus I include this single event, with a question mark, for the 500-year estimate. More certain is evidence of 3–5 severe fires in the last 2000 years (p. 42), but those all preceded 870 cal yr BP.

^5^ These authors were not focused on counting the number of fire-episodes over the last 2000 years, thus I roughly estimated this from Fig 5b in Pierce and Meyer [33] as between 7–13, as there are 7 broad peaks in this figure, but they also report 9 major debris flows between about 950 and 1150 AD, thus the total could reach as many as 13. No severe fires occurred in the last 500 years.

^6^ This author provided data on the number of watersheds, out of six sampled, that burned in high-severity events [36]. I used these data to approximate a high-severity fire rotation using the standard formula: period of observation / fraction of area burned. Thus, for the last 550 years, a total of 7 watersheds burned, thus the fire rotation is 550 / (7/6) = 471 years. And for the last 2000 years, a total of 18 watersheds burned, thus a fire rotation of 667 years. However, Bigio indicates that sample locations may be high in a watershed, thus it is not known that the whole watershed burned. This leaves these estimates as minima, which I have indicated by using “>” before the estimate.

^7^ These authors identify periods of severe-fire activity after c. 1800 cal, yr BP, a peak in 800–500 cal yr BP, and at least one large, severe fire in the last 400 years, thus perhaps 3 episodes in the last 2000 years and one in the last 500 years. However, this is my approximation from their data, as they do not report recurrence intervals for severe fire.

The phrase “FIA data” incorrectly appears three times in the section, “Comparing fire rotations in recent historical periods.”

The seventh sentence should be: “Thus, as a first approximation, I used the range of available estimates from GLO data, early historical data, and aerial photographs, which is 217–849 years (Table 1), as the standard to compare with recent rates in each region.”

The twenty-eighth sentence should be: “Using the best available data on historical rates of high-severity fire, which included estimates from GLO reconstructions, analysis of early aerial photography, and early historical data (Table 1), I classified recent high-severity fire rotations, relative to the range of historical fire rotations, which is 217–849 years (Table 1).”

The thirtieth sentence should be: “To evaluate future projected increases in high-severity fire, I compared projected high-severity fire rotations to the historical high-severity fire rotations estimated by GLO, aerial photo, and early historical data, and classified the outcomes as above (e.g., too long).”
